# Biosurfactant Produced by *Salmonella* Enteritidis SE86 Can Increase Adherence and Resistance to Sanitizers on Lettuce Leaves (*Lactuca sativa* L., *cichoraceae*)

**DOI:** 10.3389/fmicb.2016.00009

**Published:** 2016-01-22

**Authors:** Eliandra M. Rossi, Luniele Beilke, Marília Kochhann, Diana H. Sarzi, Eduardo C. Tondo

**Affiliations:** ^1^Laboratório de Microbiologia, Departamento de Ciências Biológicas e da Saúde, Universidade do Oeste de Santa CatarinaSão Miguel do Oeste, Brazil; ^2^Laboratório de Microbiologia e Controle de Alimentos, Instituto de Ciência e Tecnologia de Alimentos, Universidade Federal do Rio Grande do SulPorto Alegre, Brazil

**Keywords:** lettuce, microbial adherence and resistance, *Salmonella* Enteritidis SE86, biosurfactant, disinfection

## Abstract

*Salmonella* Enteritidis SE86 is an important foodborne pathogen in Southern Brazil and it is able to produce a biosurfactant. However, the importance of this compound for the microorganism is still unknown. This study aimed to investigate the influence of the biosurfactant produced by *S*. Enteritidis SE86 on adherence to slices of lettuce leaves and on resistance to sanitizers. First, lettuce leaves were inoculated with *S*. Enteritidis SE86 in order to determine the amount of biosurfactant produced. Subsequently, lettuce leaves were inoculated with *S*. Enteritidis SE86 with and without the biosurfactant, and the adherence and bacterial resistance to different sanitization methods were evaluated. *S*. Enteritidis SE86 produced biosurfactant after 16 h (emulsification index of 11 to 52.15 percent, *P* < 0.05) and showed greater adherence capability and resistance to sanitization methods when the compound was present. The scanning electron microscopy demonstrated that *S*. Enteritidis was able to adhere, form lumps, and invade the lettuce leaves’ stomata in the presence of the biosurfactant. Results indicated that the biosurfactant produced by *S*. Enteritidis SE86 contributed to adherence and increased resistance to sanitizers when the microorganism was present on lettuce leaves.

## Introduction

*Salmonella* Enteritidis SE86 is a recognized food pathogen responsible for several foodborne disease (FBD) outbreaks in Southern Brazil (Geimba et al., 2004; Oliveira et al., 2009; Tondo and Ritter, 2012; Capalonga et al., 2014; Tondo et al., 2015). Several studies have been carried out taking into account the importance of this pathogen with the aim of understanding the reasons that it continues to be an important foodborne pathogen in this region since 1999 (Geimba et al., 2004; Capalonga et al., 2014; Tondo et al., 2015). Among all the characteristics that may contribute to that, we may highlight its great acid adaptation capability when the pathogen is exposed to acidic environments and, as a consequence, an increase in virulence (Perez et al., 2012) and ability to survive in simulated gastric fluid, (pH 1.5; 2), besides its resistance to sodium hypochlorite at 200 and 400 ppm (Machado et al., 2010). A previous study (Machado, 2007) demonstrated that *S*. Enteritidis SE86 was able to produce expressive amounts of biosurfactant during its growth in BHI broth. Nevertheless, the characteristics and functions of this compound have not been studied yet.

The term biosurfactant is described as a “surface active agent” produced by microorganisms (Marchant and Banat, 2012). These are amphiphilic compounds used as detergents or wetting, emulsifying, dispersing, and foaming agents in many industrial formulations (Nitschke and Pastore, 2002). Even though they have been highly used, the physiological function of biosurfactants for microbial cells is still not completely understood, and the way these compounds rule food microorganisms is practically unknown. One of the few studies concerning biosurfactants in foods was published by Mellor et al. (2011), who reported that a biosurfactant produced by *Pseudomonas fluorescens* was able to alter the characteristics of chilled chicken meat (increased decomposition) and the compound facilitated the survival of the bacterium.

The production of biosurfactants is usually associated with the presence of large amounts of microorganisms (Ron and Rosenberg, 2001) and this factor can contribute to increased pathogenicity. Also, several researchers have reported that biosurfactants can contribute to the adherence of pathogens to surfaces and the formation of biofilms (Ron and Rosenberg, 1999; Ron and Rosenberg, 2001; Nitschke and Pastore, 2002). Furthermore, the ability of microorganisms to produce biosurfactants can also be linked with their resistance to sanitizers, because generally they present organic compounds that can protect bacterial cells.

Recently, it was stated that bacteria such as *S*. Enteritidis have a natural tendency to stick to surfaces, which includes lettuce leaves (Lima et al., 2013).

Lettuce (*Lactuca sativa* L., *cichoraceae*) is the most consumed green leaf in the world; this is a plant of easy acquisition, standing out due to its nutritional quality and because it is considered a low cost leafy vegetable (Abreu et al., 2010; Lima et al., 2013). During their growing cycle, lettuces can be contaminated by *Salmonella* and, as a consequence, several cases of salmonellosis have been related to the consumption of lettuces (Horby et al., 2003; Sagoo et al., 2003; Takkinen et al., 2005; Nygard et al., 2008; Irvine et al., 2009). In order to avoid contamination, lettuce leaves must be washed and sanitized before going to the table. However, if a biosurfactant is produced by *Salmonella*, microbial cells can easily adhere to the leaves and be protected against inactivation.

The aim of this study was to investigate the influence of the biosurfactant on adherence and resistance of *S*. Enteritidis SE86 to sanitizers on lettuce leaves.

## Materials and Methods

### Lettuce Samples

All lettuces used in this study were purchased in a supermarket in Porto Alegre, Capital of Rio Grande do Sul, Southern state of Brazil. Before the experiments started, lettuces were transported to the laboratory, inside thermal boxes, at 4°C for a maximum period of one hour. Before experiments, injured leaves were removed and the remaining ones were washed with potable water. Whole lettuce leaves were used for the experiments on resistance to sanitizers described in “Influence of Biosurfactant on the Efficiency of Sanitation Methods Used for Disinfection of Whole Lettuce Leaves Contaminated with *S.* Enteritidis SE86.” Slices of lettuce leaves with sizes of 10 cm × 10 cm were used for the experiments of adherence, according to Sagong et al. (2011). This was done in order to express results as number of CFU/cm^2^. All sliced leaves were cut similarly, aiming to avoid interference in the results.

Before experiments, whole lettuce leaves and sliced leaves were washed and sanitized with potable water with 200 ppm sodium hypochlorite added, for 15 min (Antunes, 2009). After that, leaves were rinsed with sterile distilled water with 0.5% sodium thiosulfate added (Synth, Diadema-SP).

### Microorganism

In this study, we used the *S*. Enteritidis SE86 strain, which was isolated from a cabbage involved with a salmonellosis outbreak in the State of Rio Grande do Sul, Brazil, in 1999. This strain was characterized by Geimba et al. (2004) and presents the same profile and genotypic characteristics of *S.* Enteritidis responsible for several cases of salmonellosis that occurred from 1999 to 2012 in Rio Grande do Sul (Capalonga et al., 2014; Tondo et al., 2015). For the tests, the strain was cultivated in BHI broth (Oxoid, Basingstoke, England) at 36 ± 1°C, for approximately 18 h.

### Biosurfactant Production on Lettuce Leaves

Four whole lettuce leaves were submerged in 100 ml of minimal medium containing 4.4 log CFU/ml of *S*. Enteritidis SE86 and incubated at 36 ± 1°C for 120 h.

Aliquots of 6 ml were removed every two hours for up to 60 and, after each time period, aliquots were withdrawn every 24 h for up to 120 h of culture in order to determine the emulsification index (IE24), pH, and bacterial count. Bacterial counts were performed in triplicate by seeding the samples onto plates containing xylose lysine deoxycholate agar (XLD: Merck, Darmstadt, Germany) and incubated at 36 ± 1°C for 24 h. The pH was evaluated by aliquots (10 ml) of the samples and then analyzed with a pH meter (PHTECK). The emulsification index (IE24) was assessed using the method described by Cooper and Goldenberg (1987).

All experiments were repeated three times and the averages were subsequently expressed as the final result.

### Preparation of the Inoculum of *S*. Enteritidis SE86 With and Without Biosurfactant

The inoculum of *S*. Enteritidis SE86 without biosurfactant was prepared using 40 ml of BHI broth (Merck, Darmstadt, Germany) incubated at 36 ± 1°C for 72 h. After incubation, the culture was centrifuged at 3500 rpm for 15 min and washed with phosphate buffered saline (PBS) three times. Then, the washed cells were inoculated in 100 ml minimal medium until they reached a concentration of approximately 8.0 log CFU/ml.

In order to prepare the inoculum of *S*. Enteritidis SE86 with biosurfactant, the compound was partially purified, according to the following procedures. The biosurfactant recovery was prepared by centrifuging at 3500 rpm for 15 min inoculum *S*. Enteritidis SE86 in BHI broth incubated at 36 ± 1°C for 72 h. The supernatant was homogenized with ethanol (–4°C) at 95% concentration 4:1 and stored at 4°C for 24 h. Subsequently, the precipitate (biosurfactant) was recovered by centrifugation at 3500 rpm for 15 min and the supernatant was discarded. After the alcohol had evaporated completely, the pellet was resuspended in sterile distilled water and dialyzed. The dialysis was done using a membrane tube (SIGMA) submitted to constant agitation in distilled water for 24 h (Kumar et al., 2004; Ciapina, 2008; Pacheco et al., 2010). Hundred milliliter of solution with partially purified biosurfactant and 8.0 log CFU/ml of *S.* Enteritidis SE86 were used.

### Resistance to Sanitizers of *S.* Enteritidis SE86 With and Without Biosurfactant *In Vitro*

The susceptibility of *S.* Enteritidis SE86 with and without biosurfactant to sanitizers *in vitro* was evaluated using sodium hypochlorite (50 and 200 ppm) and vinegar (2 and 20%). The test was performed according to the methodology recommended by Ordinance 101/93 published by the Brazilian Ministry of Agriculture and Food Supply (Brasil, 1993).

Initially, the concentrations of sodium hypochlorite (Q. Boa^®^) and vinegar (fermented acetic acid from red wine and alcohol—koller^®^) were prepared in sterile distilled water. Nine milliliter of each sanitizer were aseptically placed into sterile vials, to which was added 1 ml of bovine serum albumin solution (1%). After that, 0.1 ml inoculum (*S*. Enteritidis SE86 with and without the biosurfactant) was added separately to each tube containing sanitizers and the exposure time was measured. After 5, 10, 15, 20, and 30 min of exposure, an aliquot of 0.01 ml of suspension was transferred into tubes containing BHI broth. The tubes were incubated for 96 h at 36 ± 1°C, and the bacterial growth was checked every 24 h. In the case of bacterial growth, the test was considered positive (resistant). The negative confirmation of results (tubes without growth) was performed through inoculation on trypticase soy agar (TSA agar, Merck, Darmstadt, Germany) incubated at 36 ± 1°C for 24 h.

Each experiment was performed in triplicate on different days.

### Adherence of *S*. Enteritidis SE86 to Slices of Lettuce Leaves (*Lactuca sativa L., cichoraceae*)

Adherence of *S*. Enteritidis SE86 to slices of lettuce leaves was assessed using the methods proposed by Lima et al. (2013) with the following adaptation: the slices of lettuce leaves were cleaned, as described in Section “Lettuce Samples”.

Before each treatment, three slices of lettuce leaf were immersed in 100 ml of minimal medium containing *S*. Enteritidis SE86 at a concentration of approximately 8.0 log CFU/ml, with and without the biosurfactant, for 15, 30, and 60 min at room temperature (25°C). The preparation of the inoculum of *S.* Enteritidis SE86 with and without biosurfactant is described in Section “Preparation of the Inoculum of *S*. Enteritidis SE86 With and Without Biosurfactant”. After that, slices of lettuce leaf were submerged in 100 ml of PBS and immediately sonicated for five minutes, using ultrasonic equipment (LF Equipamentos, Anhangaba SP) with intensity of 40 kHz. Sonication was used in order to remove adhered cells following the methods described by Sinde and Carballo (2000). This method was used because it does not damage cells and is considered very efficient in removing bacteria from biomaterials, especially from rough or irregular surfaces (An and Skowronski, 2000).

The counting of *S*. Enteritidis SE86 was performed on XLD agar incubated at 36 ± 1°C for 24 h. Counts were done in triplicate and each experiment was repeated five times.

### Influence of Biosurfactant on the Efficiency of Sanitation Methods Used for Disinfection of Whole Lettuce Leaves Contaminated with *S*. Enteritidis SE86

First of all, 250 g of whole lettuce leaves were immersed into 500 ml of the *S.* Enteritidis SE86 inoculum with and without biosurfactant (prepared as described in Section “Preparation of the Inoculum of *S*. Enteritidis SE86 With and Without Biosurfactant”) for 60 min.

Sanitation treatments were performed by immersing artificially contaminated lettuce leaves (25 g) in 500 ml of each treatment solution (i.e., potable water for 30 min; 50 and 200 ppm sodium hypochlorite for 15 and 30 min; 2 and 20% vinegar aqueous solution for 15 min). At the end of the contact time, each treatment solution was drained off, and leaves were rinsed with 200 ml of neutralizing buffer solution (0.5% thiosulfate sodium, Synth, Diadema SP) for 30 s, as recommended by Abadias et al. (2008), and then rinsed with potable water.

The negative and positive controls were non-contaminated lettuce leaves and lettuce leaves artificially contaminated with *S*. Enteritidis SE86, respectively. Washing was carried out only with potable water in order to evaluate of bacterial removal.

After treatments, lettuce leaves (25 g) were blended in a Stomacher bag containing 225 ml 0.1% peptone water (Merck, Darmstadt, Germany) for 60 s. *S*. Enteritidis SE86 counting was carried out on XLD agar after incubation at 36 ± 1°C for 24 h. Typical colonies (black) were counted in triplicate and the identity of the microorganism confirmed by biochemical tests.

All treatments were performed ten times on different days and the measurement of free chlorine in solutions was done using a Spectroquant^®^ Kit (Merck).

### Scanning Electron Microscopy of *S*. Enteritidis SE86 on Surface of Lettuce Leaves With and Without Biosurfactant

Lettuce slices (1 cm × 1 cm) were prepared using the central region of lettuce leaves (washed and disinfected as described in Section “Lettuce Samples”).

Three artificially contaminated lettuce slices with and without biosurfactant were let for 1 h at room temperature (25°C). After that, leaves were gently washed twice using 0.1% peptone water and fixed with 3.0% glutaraldehyde and 0.05 M phosphate buffer, pH 7.0, for 1 h. The slices were washed four times (15 min each) with phosphate-buffered saline. After that, samples were dehydrated by increasing concentrations of ethanol solution (30, 50, 70, 80, 95, and 100%), with 15 min of contact each, and finally acetone PA for 30 min. The slices were dried with CO_2_ in a critical-point drier (CPD 030; Bal-Tec), coated with gold (BAL-TEC SCD 050), and taken for observation on a JSM 5800 scanning electron microscope (SEM). Three lettuce slices submerged only in sterile distilled water were used as negative controls.

### Statistical Analysis

The ANOVA test was applied (Assistat 7.7 Beta) with *P* < 0.05, in order to assess significant differences in the adherence of *S*. Enteritidis SE86 and its resistance to washing and disinfecting methods on lettuce leaves.

## Results

### Production of Biosurfactant on Lettuce Leaves

**FIGURE 1:**
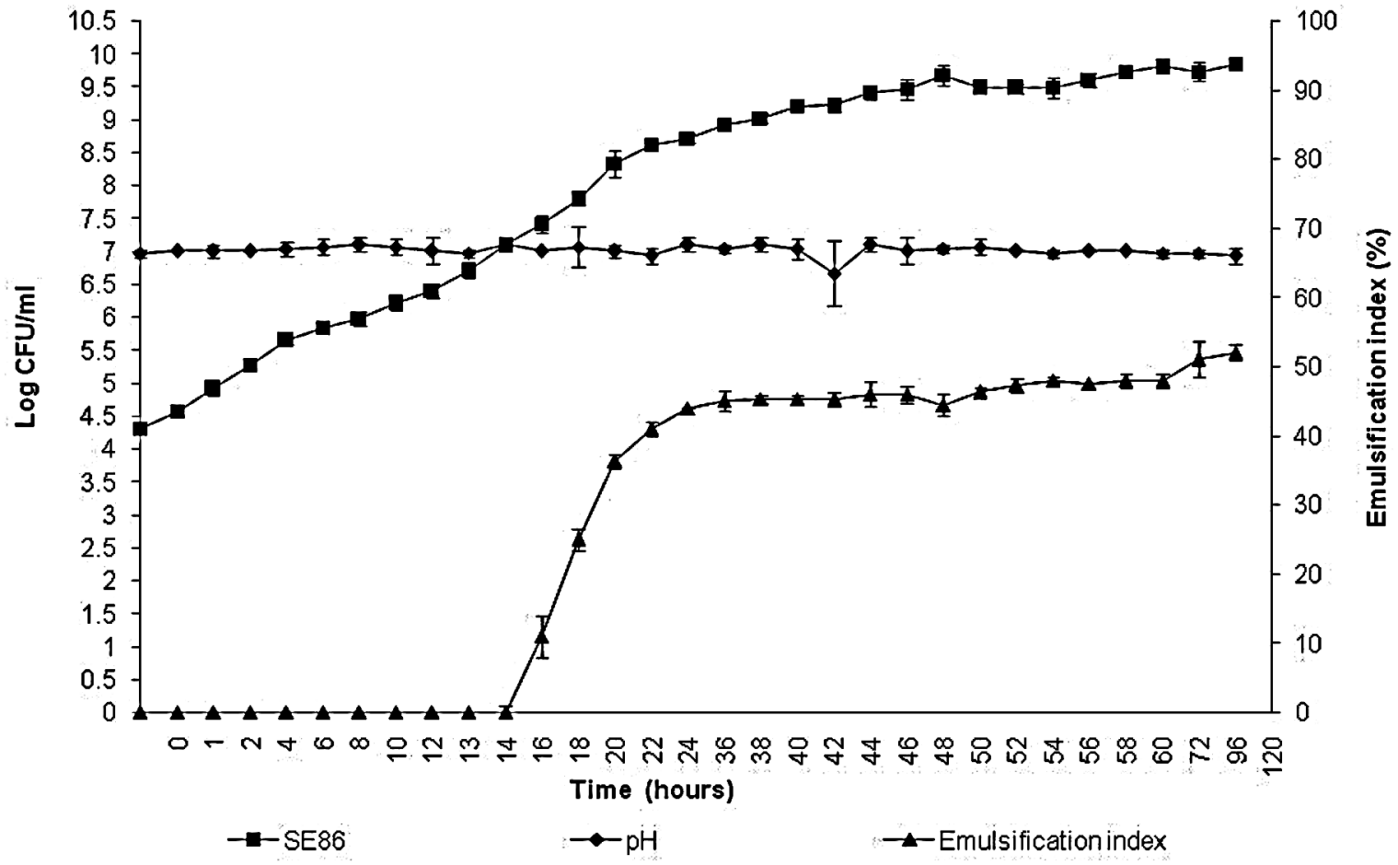
**Growth, pH, and emulsification index of *Salmonella* Enteritidis SE86 in minimal medium with whole lettuce leaves for 120 h at 36 ± 1°C**.

The results showed that *S*. Enteritidis SE86 produced biosurfactant when in contact with the lettuce leaves for more than 16 h, presenting an emulsification index (IE_24_) of 11% when bacterial population reached 7.11 log CFU/ml (**Figure 1**). The greater emulsification index was 52.15%, after 120 h of contact with lettuce leaves and when the population was 9.8 log CFU/ml and the pH remained at 7.0 during all experiments. During the preparation of the inoculum in minimal medium, SE86 also produced emulsifier (EI24 46%); however, after 120 h, the IE24 decreased to 3% (results not shown), probably because the energy sources were depleted.

### *In Vitro* Resistance to Sanitizers

The *in vitro* testing of the susceptibility to sanitizers revealed that the biosurfactant was responsible for increasing the survival of *S*. Enteritidis SE86 in 50ppm sodium hypochlorite and 2 and 20% vinegar solution. As an example, SE86 without biosurfactant was completely inactivated by 50 ppm sodium hypochlorite in 15 min, while with biosurfactant, the microorganism survived for 30 min. Similarly, SE86 without surfactant was eliminated by 2 and 20% vinegar after 15 and 0 min of exposure, respectively. Nevertheless, the presence of surfactant made SE86 survive for 20 and 5 min, respectively (**Table 1**). The biosurfactant did not influence the survival of SE86 exposed to 200 ppm sodium hypochlorite.

**Table 1:** *In vitro* susceptibility testing to disinfectants (200 and 50 ppm sodium hypochlorite and 2 and 20% vinegar solution) of *S*. Enteritidis SE86 with and without biosurfactant.

Sanitizers	Exposure time (minutes) of *S.* Enteritidis SE86 without biosurfactant					Exposure time (minutes) of *S.* Enteritidis SE86 with biosurfactant				
	5	10	15	20	30	5	10	15	20	30
200 ppm sodium hypochlorite	S	S	S	S	S	S	S	S	S	S
50 ppm sodium hypochlorite	R	R	R	S	S	R	R	R	R	R
2% vinegar solution	R	R	R	S	S	R	R	R	R	S
20% vinegar solution	S	S	S	S	S	R	S	S	S	S
Water	R	R	R	R	R	R	R	R	R	R

*R: resistant; S: sensitive.*

### Influence of Surfactant on the Adherence of *S*. Enteritidis SE86 to Slices of Lettuce Leaf

The results of this study showed that there were significant differences (*P* < 0.05) in the adherence of *S*. Enteritidis SE86 to slices of lettuce leaf when biosurfactant was present (**Table 2**). The highest counts of adhered SE86 were observed after 60 min of contact with slices of lettuce leaf. At that time, average counts of 7.3 log CFU/cm^2^ and 4.1 log CFU/cm^2^ were obtained on lettuce leaves with and without biosurfactant, respectively.

**Table 2:** Median and standard deviation about the adherence of *S*. Enteritidis SE86 with and without biosurfactant on slices of lettuce leaves at different times.

Time (minutes)	*S*. Enteritidis SE86 without biosurfactant (log CFU/cm^2^)	*S*. Enteritidis SE86 with biosurfactant (log CFU/cm^2^)
15	3.5 ± 0.3^c^	6.3 ± 0.2^b^
30	3.5 ± 0.3^c^	6.0 ± 0.7^b^
60	4.1 ± 0.5^c^	7.3 ± 0.3^a^

*Values represent the means of five replicates. Different letters represent significant differences (P < 0.05).*

**FIGURE 2:**
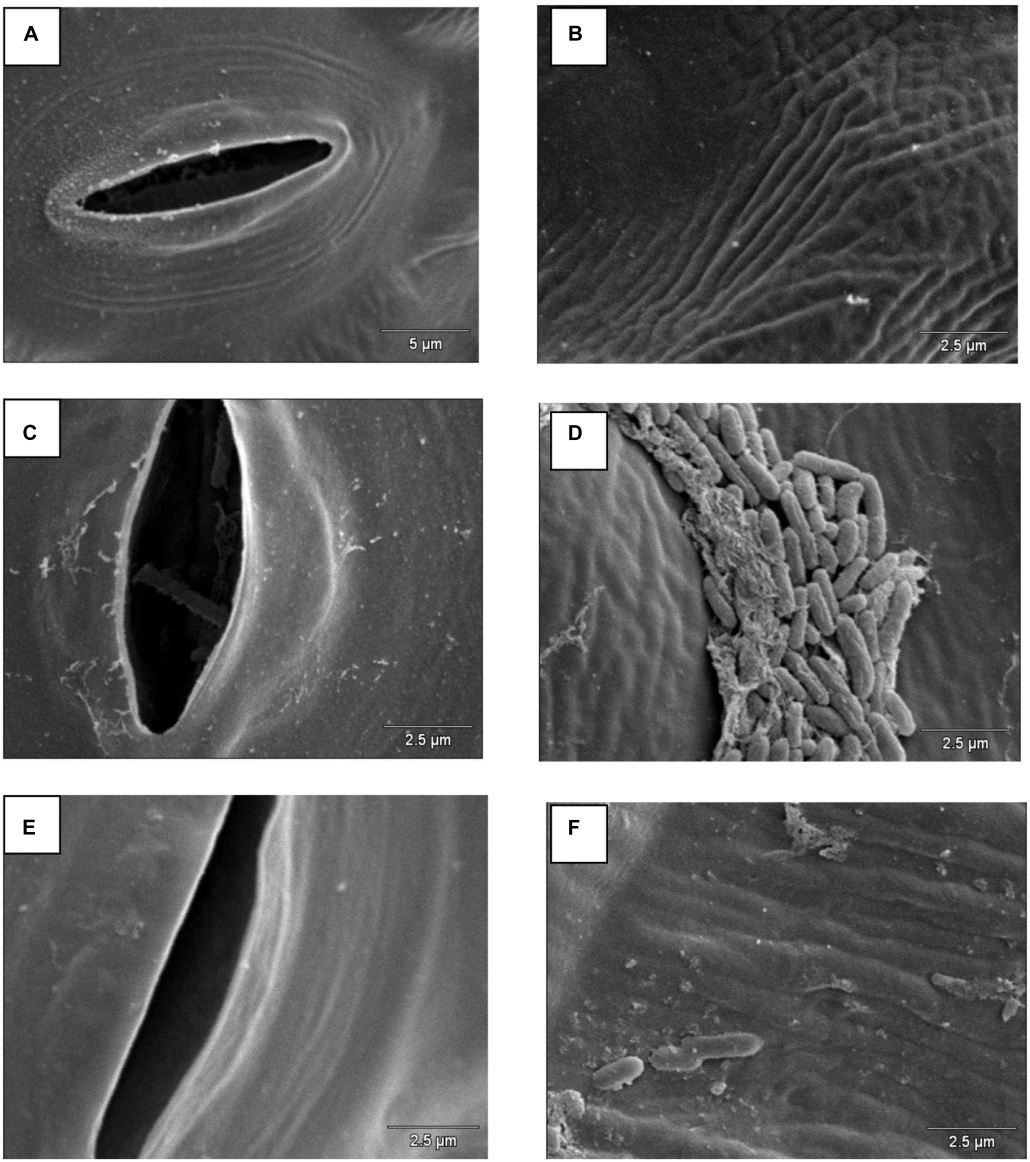
**Scanning electron microscopy on lettuce leaf surface infected with *S.* Enteritidis SE86 with and without biosurfactant.** Negative control: **(A)** stomata and **(B)** lettuce leaf surface. *S.* Enteritidis SE86 with biosurfactant: **(C)** *S*. Enteritidis SE86 inside a stomata, and **(D)** lumps of *S*. Enteritidis SE86 on lettuce leaf surface. *S*. Enteritidis SE86 without biosurfactant: **(E)** stomata without *S*. Enteritidis SE86 and **(F)** lettuce leaf surface without formation lumps of *S*. Enteritidis SE86.

Scanning electron microscopy demonstrated that *S*. Enteritidis SE86 was able to adhere to the slices of lettuce leaf, forms lumps, and enter the stomata when the biosurfactant was present (**Figure 2**).

### Resistance to Sanitation Methods of Lettuce Leaves

**FIGURE 3:**
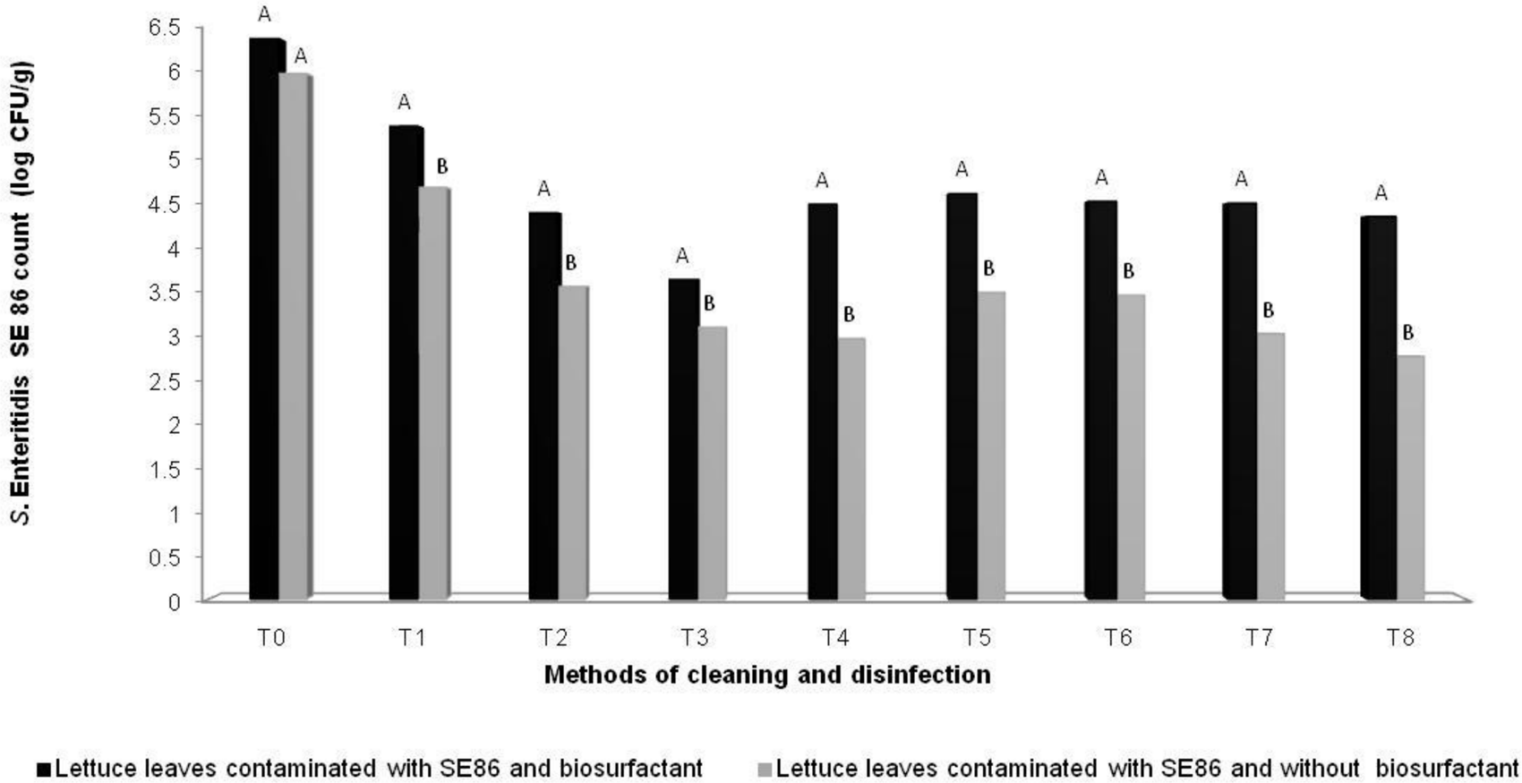
**Mean (log CFU/g) of *S*. Enteritidis SE86 on whole lettuce leaves contaminated with *S*. Enteritidis SE86 and biosurfactant (Experiment 1) and *S*. Enteritidis SE86 without biosurfactant (Experiment 2).** ^∗^Statistical analysis between Experiments 1 and 2. Different letters indicate significant differences (*P* < 0.05). T0: Control positive: lettuce leaves contaminated with *S*. Enteritidis SE86; T1: washing lettuce with potable water; T2: immersion in potable water for 30 min; T3: immersion in 200 ppm of sodium hypochlorite for 15 min; T4: immersion in 200 ppm sodium hypochlorite for 30 min; T5: immersion in 2% vinegar solution for 15 min; T6: immersion in 20% vinegar solution for 15 min; T7: immersion in 50 ppm of sodium hypochlorite for 15 min; T8: immersion in 50ppm of sodium hypochlorite for 30 min.

It was observed that all treatments reduced the amount of *S*. Enteritidis SE86 on lettuces, but lettuces contaminated with *S*. Enteritidis SE86 and with biosurfactant demonstrated higher numbers of survival cells (significant difference *P* < 0.05) than lettuces contaminated with *S*. Enteritidis SE86 without the surfactant (**Figure 3**). Reductions in counts of *S*. Enteritidis SE86 with biosurfactant ranged from 1.0 to 2.8 log CFU/g, whereas the reductions of *S*. Enteritidis SE86 without biosurfactant ranged from 1.3 to 3.3 log CFU/g (*P* < 0.05) (**Table 3**). It was observed that the most effective treatment of lettuce contaminated with *S*. Enteritidis SE86 and biosurfactant was washing it with potable water and submerging it in 200 ppm of sodium hypochlorite for 15 min. This showed a reduction of 2.8 log CFU/g. However, when lettuce leaves were contaminated only with *S*. Enteritidis SE86, the most effective reduction (3.3 log CFU/g) was obtained by the treatment that washed leaves with potable water and sanitized them with 50 ppm sodium hypochlorite for 30 min. This result showed that *S*. Enteritidis SE86 without biosurfactant was inactivated by lower concentrations of sodium hypochlorite.

**Table 3:** Reduction (log CFU/g) in counts of *S*. Enteritidis SE86 with and without biosurfactant on whole lettuce leaves after the treatments were performed.

Treatments	Reduction in *S.* Enteritidis SE86 counts on lettuce (log CFU/g) with biosurfactant	Reductions in *S.* Enteritidis SE86 counts on lettuce (log CFU/g) without biosurfactant
Washing with water	1.0^b^	1.3^b^
Water (30 min)^∗^	2.0^c^	2.5^c^
200 ppm sodium hypochlorite (15 min)^∗^	2.8^d^	2.9^c,d^
200 ppm sodium hypochlorite (30 min)^∗^	1.9^c^	3.1^c,d^
2% vinegar solution (15 min)^∗^	1.8^c^	2.5^c,d^
20% vinegar solution (15 min)^∗^	1.9^c^	2.6^c,d^
50 ppm sodium hypochlorite (15 min)^∗^	1.9^c^	3.0^c,d^
50 ppm sodium hypochlorite (30 min)^∗^	2.1^c^	3.3^d^

*Log CFU/g: colony forming unit/g converted to log_10_. ^∗^The period of time the solutions spent submerged is shown in parenthesis next to each treatment used. Statistical analysis between treatments. Different letters indicate statistically significant differences between treatments (P < 0.05).*

*Salmonella* Enteritidis SE86 with biosurfactant was more resistant on lettuce leaves than in *in vitro* tests (**Table 1**; **Figure 3**).

## Discussion

Microorganisms develop survival abilities in different environments and biosurfactant production can be an advantage to survive in foods (Mellor et al., 2011). However, the exact physiological function of biosurfactants is not yet completely elucidated (Nitschke and Pastore, 2002; Hamme et al., 2006; Abdel-Mawgoud et al., 2009; Jirku et al., 2015).

Results of the present study demonstrated that *S*. Enteritidis SE86 was able to produce biosurfactant on lettuce leaves and this ability may have facilitated the access to cutin on lettuce leaves, one functional component of the cuticle deposited on the surfaces and within the epidermal walls of aerial parts of plants. Cutin is composed of three dimensional polyesters of long fatty acid chains (Bacic et al., 1988) and the amphipathic property of biosurfactant may facilitate access to nutrients present on lettuce leaves, supplying energy for bacterial growth.

Several research groups have reported that environmental microorganisms are able to produce biosurfactants (Chen et al., 2012; Jain et al., 2013; Ayed et al., 2014; Maoa et al., 2015; Rosa et al., 2015); however, there are no scientific reports showing the production of surfactants by foodborne pathogens. To our knowledge, the present study is the first that demonstrates biosurfactant production by *Salmonella*. Other reports have demonstrated the production of biosurfactants by degradative microorganisms on foods. For example, according to Mellor et al. (2011), the biosurfactant produced by *Pseudomonas fluorescens* contributed to increasing the total bacterial count on chicken stored aerobically for three days, suggesting that the biosurfactant contributed to the bioavailability of nutrients for the bacteria. These researchers suggested that the biosurfactant becomes a competitive advantage for the microorganism to maintain their survival, thereby enhancing the decomposition of chicken meat. Shaheen et al. (2010) have reported that a type of biosurfactant called surfactin may have contributed to the formation of biofilms by *Bacillus cereus* inside milk tanks.

The ability of *Salmonella* to adhere to lettuce leaves was reported by several studies (Wei et al., 2006; Patel and Sharma, 2010; Kroupitski et al., 2011; Lima et al., 2013). Results similar to the ones obtained in this study were found by Kroupitski et al. (2011), who found 7.0 log CFU of *S.* Typhimurium on the central region of lettuce leaves. Also, Lima et al. (2013) demonstrated that the count of *S.* Enteritidis cells that adhered to lettuce leaves differed *(P* < 0.05*)* between the hydroponic and conventional systems, reaching 5.2 ± 0.56 and 4.6 ± 0.26, respectively.

The influence of biosurfactants on bacterial adherence to surfaces has been quite well studied, and the results are variable. Hassan and Frank (2003) stated that Tween 85 surfactant reduced the adherence of *Escherichia coli* O157:H7 to lettuce leaves. Other researchers (Sotirova and Vasileva-Tonkova, 2009) reported that *Pseudomonas aeruginosa* NBIMCC 1390 with rhamnolipid biosurfactant increased cell hydrophobicity to 31% adherence and that these compounds caused changes in the bacterial cell surface.

The results of our study suggest that the biosurfactant contributed to increase the survival of *S*. Enteritidis SE86 on lettuce leaves. According to Wei et al. (2006), high surface adherence of bacterial populations is a competitive tool against other microorganisms.

The results of *in vitro* resistance to sanitizers and resistance to sanitation methods of lettuce leaves showed that *S*. Enteritidis SE86 with biosurfactant is more resistant to antimicrobial activity of the compounds tested.

The bactericidal action of sodium hypochlorite is the result of microbial cell oxidation, after contact of sanitizer and cells (Watters et al., 2002; Møretrø et al., 2012; Bermúdez-Aguirre and Barbosa-Cánovas, 2013). According to Møretrø et al. (2012), pH and the presence of organic matter can affect the antimicrobial action of sodium hypochlorite. In our study, it was observed that biosurfactant decreased the antimicrobial action of sodium hypochlorite, probably because this organic compound linked to the sanitizer or avoided the contact of cells with the sanitizer.

Some studies have shown that acetic acid (vinegar) can reduce the amount of bacteria on foods and surfaces, including whole lettuce leaves (Karapinar and Gonul, 1992; Oliveira et al., 2012). Our study showed that *S*. Enteritidis SE86 in the presence of biosurfactant was more resistant to both vinegar concentrations (solution 2 and 20%). The counts of *S*. Enteritidis SE86 with biosurfactant on whole lettuce leaves sanitized with vinegar solution showed a reduction of 1.8 and 1.9 log CFU/g, whereas the reductions of *S*. Enteritidis SE86 without biosurfactant were 2.5 and 2.6 log CFU/g (**Table 3**).

Vinegars are able to decrease the external and internal pH of cells, inactivating microbial enzymes, and damaging membrane function and metabolic activities such as the transport of nutrients (Chang and Fang, 2007; Ölmez and Kretzschmar, 2009). The less effective antimicrobial action of vinegar solution on lettuce contaminated with *S*. Enteritidis SE86 in the presence of biosurfactant suggested that biosurfactant protected SE86 from contact with the vinegar solution, or this compound was able to neutralize pH action.

The greater resistance of *S*. Enteritidis SE86 in the presence of biosurfactant on lettuce leaves suggests that the biosurfactant production may be a mechanism used by the bacterium to maintain its survival in different environments.

Thus this study demonstrated that *S*. Enteritidis SE86 can use the biosurfactant to increase its adhesion to the surface of lettuce leaves, form lumps, and also to penetrate the stomata of lettuce leaves. These effects may influence the increase of resistance to vinegar and sodium hypochlorite during lettuce sanitization. Furthermore, the surfactant production by adhered cells may protect them, avoiding contact with sanitizers.

## Conclusion

Based on the results found during this study, it can be concluded that high counts of *S.* Enteritidis SE86 were able to produce biosurfactant on lettuce leaves. The presence of biosurfactant S. Enteritidis SE86 increased the adherence to slices of lettuce leaf and decreased the antimicrobial action of sanitizers (vinegar and sodium hypochlorite) used to sanitize whole lettuce leaves. In addition, when SE86 was added with biosurfactant and was analyzed by scanning electron microscopy, lumps of cells were observed and the bacterium was able to enter the stomata. The same results were not observed in the absence of biosurfactant.

New studies are necessary to investigate other probable functions of biosurfactant produced by SE86. As a perspective of the present study, we suggest investigating the influence of this biosurfactant on the microbial ecology of lettuce leaves, and on the multiplication and survival of SE86 in other foods.

## Author Contributions

ER: contributes to the development of experimental research, data analysis, and preparation of the article. LB: contributes to the development of experimental research. MK: contributes to the development of experimental research. DS: contributes to the development of experimental research. ET: contributes to the data analysis and preparation of the article.

## Conflict of Interest Statement

The authors declare that the research was conducted in the absence of any commercial or financial relationships that could be construed as a potential conflict of interest.
